# Sensor Data Fusion for Accurate Cloud Presence Prediction Using Dempster-Shafer Evidence Theory

**DOI:** 10.3390/s101009384

**Published:** 2010-10-18

**Authors:** Jiaming Li, Suhuai Luo, Jesse S. Jin

**Affiliations:** 1 CSIRO ICT Centre, Corner of Vimiera and Pembroke Roads, Marsfield, NSW 2122; Australia; 2 The University of Newcastle, University Drive, Callaghan, NSW 2308, Australia; E-Mails; Suhuai.Luo@newcastle.edu.au (S.H.L.); Jesse.Jin@newcastle.edu.au (J.S.J.)

**Keywords:** multi-sensor, data fusion, dempster-shafer, prediction, renewable energy, virtual power station

## Abstract

Sensor data fusion technology can be used to best extract useful information from multiple sensor observations. It has been widely applied in various applications such as target tracking, surveillance, robot navigation, signal and image processing. This paper introduces a novel data fusion approach in a multiple radiation sensor environment using Dempster-Shafer evidence theory. The methodology is used to predict cloud presence based on the inputs of radiation sensors. Different radiation data have been used for the cloud prediction. The potential application areas of the algorithm include renewable power for virtual power station where the prediction of cloud presence is the most challenging issue for its photovoltaic output. The algorithm is validated by comparing the predicted cloud presence with the corresponding sunshine occurrence data that were recorded as the benchmark. Our experiments have indicated that comparing to the approaches using individual sensors, the proposed data fusion approach can increase correct rate of cloud prediction by ten percent, and decrease unknown rate of cloud prediction by twenty three percent.

## 1. Introduction

Multi-sensor data fusion [1,2] has been developed recently to solve a diverse set of problems having common characteristics. It is analogous to the ongoing cognitive process used by humans to integrate data continually from their sensors to make inferences about the external world. Humans receive and process sensory data including sights, sounds, smells, tastes and touch, which are then assessed to draw conclusions about the environment and what they mean. Data fusion is an important tool for improving the performance of detecting system when various sensors are available. It seeks to combine data from multiple sensors to perform inferences that will be more efficient and potentially more accurate than if they were achieved by means of a single sensor. Fusion of multi-sensor data provides significant advantages over single source data in two aspects: one is the statistical advantage gained by combining data of same source (e.g., obtaining an improved estimate of a physical phenomenon via redundant observations), the other is the use of multiple types of sensors to increase the accuracy with which a quantity can be observed and characterized.

This paper introduces a novel data fusion approach based on Dempster-Shafer evidence theory [3,4]. The approach is used for cloud presence prediction in Virtual Power Station (VPS) [5], where individual small-scale renewable energy sites are aggregated together to form a “virtual” power station that appears as a single dispatchable quantity to the wider electricity system. Since such a quantity has greater benefit to the wider system than the individual responses of many uncoordinated energy sites, the virtual power station can improve the payback period for renewable energy systems. The concept relies on sophisticated prediction and aggregation mechanisms to firstly anticipate the power available from a renewable energy system, and then aggregate many small systems into one quantity with reliable output. A great challenge in fulfilling the task is to precisely predict the output of each individual solar or photovoltaic (PV) generator, which is affected by a lot of factors. Among these factors, cloud presence is the most important. Conventionally, the solutions for compensating this inaccurate prediction include using battery and dumping power. They both increase system cost. The best way is to improve the prediction accuracy of PV output. It is known that PVs in VPS are geographical distributed. If the cloud presence for near future can be accurately predict based on some available indirectly related sensor data, the PV output can be predicted accordingly. In this way, other corresponding PVs can accordingly adjust their power commitment to the grid, finally decreasing the total system cost and increasing power commitment reliability. This paper concentrates on the discussion of our approach of cloud prediction based on a group of sensor data including shortwave radiation and reflected shortwave radiation.

This paper is organized as follows. Section 2 gives a brief introduction of Dempster-Shafer theory for multi sensor data fusion. Section 3 focuses on its applications and presents our implementation for cloud prediction. Section 4 describes a series of experiments and related results to quantify the performance of cloud prediction. Finally, a conclusion is drawn in Section 5.

## 2. Dempster-Shafer Data Fusion Theory

Dempster-Shafer evidence theory offers an alternative to traditional probabilistic theory for the mathematical representation of uncertainty. It has been widely applied in various applications such as target tracking, surveillance, robot navigation, signal and image processing [6–9]. The significant innovation of Dempster-Shafer theory is that it deals with measures of “belief”, and is based on the non-classical idea of “mass” as opposed to probability. Dempster-Shafer theory does not require an assumption regarding the probability of the individual constituents of the set or interval. It has a unique advantage of making inferences from incomplete and uncertain knowledge. This is a potentially valuable tool for the evaluation of risk and reliability in engineering applications when it is not possible to obtain a precise measurement from experiments, or when knowledge is obtained from expert elicitation. An important aspect of this theory is the combination of evidence obtained from multiple sources and the modelling of conflict between them. It allows other alternative scenarios for the system, such as “unknown”.

### 2.1. Prior Requirements for Dempster-Shafer Theory

Comparing to the Bayesian theory [6] which requires prior probabilities, Dempster-Shafer theory requires some preliminary assignment of masses that reflects our initial knowledge of the system, including the “unknown” state. The key concept is basic probability assignment or mass assignment.

Basic probability assignment, represented by *m*, is a basic measure representing the support for, or confidence in, a hypothesis. If *θ* is the frame of discernment, then the mapping:

$$m:2^{\theta }\to [0,1]$$

is called basic probability assignment (BPA), if and only if it satisfies:

(1) $$\left\{ \begin{array}{l} m(\Phi )=0\\ \sum_{H\in 2^{\theta }}m(H)=1 \end{array}\right.$$

where Φ is empty hypothesis, *i.e*., nothing is happening; H is a hypothesis.

It should be emphasized that the BPA is not in general a Bayesian probability. BPA *m*(*H*) is an expression of the level of confidence exactly in a specific hypothesis H. It does not include the confidence in any particular subset of that hypothesis. For example, in a four-hypothesis frame of discernment {*ω*_1_, *ω*_2_, *ω*_3_, *ω*_4_}, statement *m*(*ω*_1_*ω*_3_) = 0.8 describes only the amount of confidence in that either the hypothesis *ω*_1_ or hypothesis *ω*_3_ is true; it does not imply any specific support measure value for *ω*_1_ or *ω*_3_ alone.

For typical Bayesian approaches, an assignment of probability to a specific hypothesis implies the amount of probability assigned to its negation, *i.e.*:

(2) $$\text{If}\;p(H)=q,\;\mathrm{then}\;p(\bar{H})=1-q$$

Whereas for Dempster-Shafer approaches, the commitment of a BPA mass to a hypothesis does not imply commitment to the remaining mass to its negation, *i.e.*:

(3) $$m(H)=\mathrm{q doesnot imply m}(\bar{H})=1-q$$

The elements of the power set can be taken to represent propositions that one might be interested in, by containing all and only the states in which this proposition is true.

### 2.2. Rules for the Combination of Evidence—Dempster’s Rule

The purpose of aggregation of information is to meaningfully summarize and simplify a corpus of data whether the data is coming from a single source or multiple sources. Familiar examples of aggregation techniques include arithmetic averages, geometric averages, harmonic averages, maximum values, and minimum values. Combination rules are the special types of aggregation methods for data obtained from *multiple* sources. These multiple sources provide different assessments for the same frame of discernment. Dempster-Shafer theory is based on the assumption that these sources are independent.

Dempster-Shafer theory gives a rule for calculating the confidence measure of each state, based on data from different evidences. Dempster’s rule of combination has been used as sensor fusion strategy, as given in equations (4) and (5).

For two sensors:

(4) $$m^{1,2}(C)=\frac{\sum_{A\cap B=C,C\neq \Phi }m^{1}(A)m^{2}(B)}{1-k}$$

For three sensors:

(5) $$m^{1,2,3}(D)=\frac{\sum_{A\cap B\cap C=D}m^{1}(A)m^{2}(B)m^{3}(C)}{1-k}$$

where $k=\sum_{A\cap B=\Phi }m^{1}(A)m^{2}(B)$ for two sensors and $k=\sum_{A\cap B\cap C=\Phi }m^{1}(A)m^{2}(B)m^{3}(C)$ for three sensors; *k* represents basic probability mass associated with conflict, which is determined by summarising the products of the BPA’s of all sets where the intersection is null. C is the intersection of states A and B in Equation (4), and D is the intersection of states A, B and C in Equation (5); *m*^1,2^ (*C*) is the new evidence updated by the evidence sources *m*^1^ (*A*) from sensor 1 and *m*^2^ (*B*) from sensor 2; and *m*^1,2,3^ (*D*) is the new evidence updated by the evidence sources *m*^1^ (*A*) from sensor 1, *m*^2^ (*B*) from sensor 2 and *m*^3^ (*C*) from sensor 3.

Unlike Bayes theory, Dempster-Shafer theory explicitly allows for an undecided state of knowledge. It can sometimes be far safer to be undecided about what a target is, than to decide wrongly and act accordingly with what might be disastrous consequences.

### 2.3. Support and Plausibility

Dempster-Shafer theory contains two new ideas that are foreign to Bayes theory. These are the notions of support and plausibility as described in equations (6) and (7) below.

(6) $$\mathrm{spt}(A)=\sum_{B|B\subseteq A}m(B)$$

(7) $$\mathrm{pls}(A)=\sum_{B|A\cap B\neq \Phi }m(B)$$

The support for the target “A”, *spt*(A), is defined as the total mass of all states implying the “A” state. Plausibility, *pls*(A), is defined as the total mass of all states that don’t contradict the “A” state. The quantity *spt*(A) can be interpreted as a global measure of one’s belief that hypothesis A is true, while *pls*(A) can be viewed as the amount of belief that could potentially be placed in A, if further information became available. The support is a kind of loose lower limit to the uncertainty. On the other hand, a loose upper limit to the uncertainty is the plausibility.

In addition to deriving these measures from the basic probability assignment (*m*), these two measures can be derived from each other. For example, *plausibility* can be derived from *support* in the following way:

(8) $$\mathrm{pls}(A)=1-\mathrm{spt}(\bar{A})$$

where *A̅* is the classical complement of *A*. This definition of plausibility in terms of belief comes from the fact that all basic assignments must sum up to 1.

## 3. Cloud Presence Prediction Using Dempster-Shafer Evidence Theory

This section will focus on Dempster-Shafer evidence theory applications in multiple sensor environments and present our implementation for the cloud presence prediction.

Let *θ* = {*cloud, sunshine*} be the set of local elements that can be observed by each sensor. The power set of *θ* denoted as 2*^θ^* is the set of all possible sub-sets of *θ*, including the empty set Φ:

(9) $$2^{\theta }=\{\Phi ,\{\mathrm{cloud}\},\{\mathrm{sunshine}\},\{\mathrm{unknow}\}\}$$

where {*unknown*} = {*cloud*} ∪ {*sunshine*}.

### 3.1. Basic Probability Assignment

Basic probability assignment is also called basic belief mass. It is the prior knowledge we have for the sensors. Currently, the sensor gives radiation output, in which cloud or sunshine information is hidden. Radiation outputs from different sensors have different responses to the cloud presence. For example, when cloud occurs, some radiation outputs may drop down, some may not, or may drop down at different amount. Basic belief masses for each local sensor are defined as follows.

#### Cloud mass

Cloud mass is defined as correct cloud prediction rate as Equation (10).

(10) $$m^{i}(c)=\frac{K_{cc}^{i}}{N_{tc}^{i}}$$

where *m^i^*(*c*) is cloud belief mass from sensor *i*; *N^i^_tc_* is the total number of cloud prediction from sensor *i*; *K^i^_cc_* is the number of correct cloud prediction from sensor *i*.

#### Sunshine mass

Sunshine mass is defined as correct sunshine prediction rate as Equation (11).

(11) $$m^{i}(s)=\frac{K_{sc}^{i}}{K_{ts}^{i}}$$

where *m^i^*(*s*) is sunshine belief mass from sensor *i*; *N^i^_ts_* is the total number of sunshine prediction from sensor *i*; *K^i^_sc_* is the number of correct sunshine prediction from sensor *i*.

#### Unknown mass

Unknown mass is defined as wrong cloud and sunshine prediction rate as Equation (12).

(12) $$m^{i}(u)=\frac{K_{\mathrm{cw}}^{i}+K_{\mathrm{sw}}^{i}}{N_{\mathrm{tc}}^{i}+N_{\mathrm{ts}}^{i}}$$

where *m^i^*(*u*) is unknown belief mass from sensor *i*; *K^i^_sw_* is the number of wrong cloud prediction from sensor *i*; *K^i^_sw_* is the number of wrong sunshine prediction from sensor *i*.

In order to satisfy condition of Equation (1), each mass is finally normalized.

### 3.2. System Diagram of Dempster-Shafer Data Fusion for Cloud Presence Prediction

**Figure 1.**
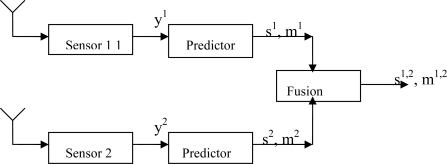
General diagram of Dempster-Shafer data fusion for two sensors.

Figure 1 shows a general diagram of our Dempster-Shafer data fusion system for cloud presence prediction. The system is consisted of two channels. Sensor 1 firstly generates output y^1^, which is the global shortwave radiation. y^1^ is sent to predictor to predict the evidence s^1^, such as cloud or sunshine, with a certain belief mass m^1^.

At the same time, sensor 2 generates output y^2^, which is the reflected shortwave radiation. y^2^ is sent to predictor to predict the evidence s^2^ with a certain belief mass m^2^. Using Dempster-Shafer rule as Equation (4), the fused evidence s^1,2^ with belief mass m^1,2^ can be derived. Note the system output is the fused evidence which could be either cloud or sunshine with certain belief mass.

## 4. Experiments and Results

A series of experiments have been designed to validate the performance of the proposed cloud presence prediction algorithm. The sensor data used come from the Automatic Weather Station of Macquarie University [10]. The data, which are sampled in one minute interval, include sunshine duration information indicating cloud presence, and different radiation data, such as global shortwave radiation, diffuse shortwave radiation and reflected shortwave radiation, *etc.* Different radiation data have different responses to the cloud presence. Based on each sensor’s output, each state, such as cloud or not, can be predicted with a certain amount of confidence. Then Dempster-Shafer fusion is used to combine the evidences to generate fused evidence.

In our experiment, global shortwave radiation data and reflected shortwave radiation data from Automatic Weather Station of Macquarie University are used to predict cloud presence. Global shortwave radiation is the incident shortwave radiation and comprises the direct and diffuse components. It is measured using a Middleton EPO7 Solarimeter. Reflected shortwave radiation is the shortwave radiation coming from the surface of the earth. It is related directly to the global shortwave radiation and the surface albedo. Reflected radiation is measured using a Middleton EPO7 Solarimeter. Sunshine duration data are used as benchmark to test the prediction accuracy. Sunshine duration is a measure of the percentage of bright sunshine observed. It is related to the duration and intensity of direct solar radiation as opposed to diffuse radiation and gives an indication of the presence of cloud. It is measured using a RS-4 Sunshine Duration Detector.

### 4.1. Cloud Presence Predictor

**Figure 2.**
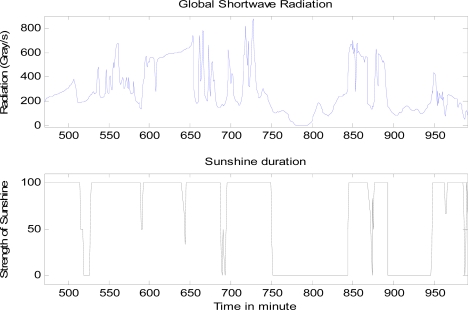
Relationship between sunshine presence and the amount of global shortwave radiation. Top: the amount of global shortwave radiation along the time. Bottom: the sunshine strength along the time.

It is observed that some certain intrinsic relationship exists between sunshine presence which is the opposite of cloud presence, and the amount of radiations detected by the sensors. Figure 2 shows an example of global shortwave radiation and corresponding sunshine duration. In the figure, the amount of radiation is measured in gray, which is the absorption of one joule of energy by one kilogram of matter in the form of ionizing radiation. From the figure, it can be seen that when sunshine drops down, *i.e.*, cloud occurs up in the sky, the strength of global shortwave radiation responds accordingly. However, the exact relationship between sunshine presence and the strength of the radiation is not clear—the amount of radiation changes may vary even for the same amount of sunshine strength. How to find out such intrinsic relationship and predict the cloud presence from the radiation sensor data is the essential task to be discussed. To investigate the performance of various approaches, three predictors were designed and tested in the cloud presence prediction system as depicted in Figure 1. These predictors are described in this section.

#### Predictor 1

The cloud and sunshine is predicted by a simple threshold as Equation (13):

(13) $$S(t)=\text{min}(\frac{X(t)}{X_{\text{max}}},\delta )/\delta$$

where *X*(*t*) is the output of sensor at time t; *X*_max_ is the maximum of *X*(*t*); *δ* is prediction threshold. Cloud is predicted when *S*(*t*) < 1, sunshine is predicted when *S*(*t*) = 1.

#### Predictor 2

Using time information in predicting cloud occurrence, the cloud is predicted only when both the original and the shifted data are less than the threshold. The prediction is expressed as Equation (14):

(14) $$S(t)=\left\{\text{min}(\frac{X(t)}{X_{\text{max}}},\delta )/\delta \right\}\times \left\{\text{min}(\frac{X(t+\tau )}{X_{\text{max}}},\delta )/\delta \right\}$$

where *X*(*t* + *τ*) is the *X*(*t*) with time-shifting of −τ. Cloud is predicted when *S*(*t*) < 1, sunshine is predicted when *S*(*t*) = 1.

#### Predictor 3

The prediction is expressed as Equation (15):

(15) $$S(t)=\left\{\text{min}(\frac{X(t)}{X_{\text{max}}},\delta )/\delta \right\}\times \left\{\text{min}(\frac{X(t+\tau )}{X_{\text{max}}},\delta )/\delta \right\}\times \left\{\text{min}(\frac{X(t-\tau )}{X_{\text{max}}},\delta )/\delta \right\}$$

where *X*(*t* + *τ*) and *X*(*t* − *τ*) are the *X*(*t*) with time-shifting of −τ and τ respectively. Cloud is predicted when *S*(*t*) < 1, sunshine is predicted when *S*(*t*) = 1.

### 4.2. Learning of Basic Belief Mass

The data used for basic belief mass learning is sunshine duration data, global shortwave radiation and reflected shortwave radiation from date 15th to 18th of April 2008. Basic belief mass is learned as equations (10), (11) and (12).

### 4.3. Dempster-Shafer Fusion

Consider two sensor inputs, of which the class set includes (c, s, u), representing cloud, sunshine and unknown state respectively. Suppose the first and second sensors provide basic belief mass as follows: m^1^(c) = 0.2, m^1^(s) = 0.6, m^2^(c) = 0.5, m^2^(s) = 0.4 and their unknown mass is m^1^(u) = 0.2, m^2^(u) = 0.1. Table 1 shows the set intersections of all hypotheses specified for this case, along with their corresponding basic belief mass products.

In Table 1, the empty intersection is shown in bold numbers, cloud intersection is shown in bold italic numbers, sunshine intersection is shown in normal numbers and unknown or ignorance is shown in underlined bold numbers. Based on Dempster’s combination rule expressed as Equation (4), the fused belief mass for cloud, sunshine and unknown can be derived as equations (16–18) respectively. In these equations, the fused cloud belief mass m^1,2^(c) will sum the terms in Table 1 in bold italic numbers and then divide by one minus the sum of the terms in bold number as shown in Equation (16). The fused sunshine belief mass m^1,2^(s) will sum the terms in Table 1 in normal numbers and then divide by one minus the sum of the terms in bold number as shown in Equation (17). The fused unknown mass m^1,2^(u) will sum the terms in Table 1 in underlined bold numbers and then divided by one minus the sum of the terms in bold number as shown in Equation (18):

(16) $$m^{1,2}(c)=\frac{0.1+0.1+0.02}{1-(0.3+0.08)}=0.355$$

(17) $$m^{1,2}(s)=\frac{0.24+0.08+0.06}{1-(0.3+0.08)}=0.613$$

(18) $$m^{1,2}(u)=\frac{0.02}{1-(0.3+0.08)}=0.032$$

### 4.4. Results of Dempster-Shafer Fusion

The test sensor data used for cloud prediction is sunshine duration data, global shortwave radiation and reflected shortwave radiation on 13th, 14th, 29th, and 30th of April, 2008. The data are exclusive from the learning data. Time shifting parameter τ is set to 2 minutes for predictor 2 and 1 minute for predictor 3.

Experiments are designed to compare the cloud prediction performance of the sensors without data fusion with that of the sensors with data fusion. Tables 2 shows the correct rate of cloud prediction (*C_c_*) and unknown rate (*U*) for individual sensors without data fusion, under different predictors. Whereas, tables 3 shows the correct rate of cloud prediction and unknown rate for the sensors with Dempster-Shafer data fusion.

**Figure 3.**
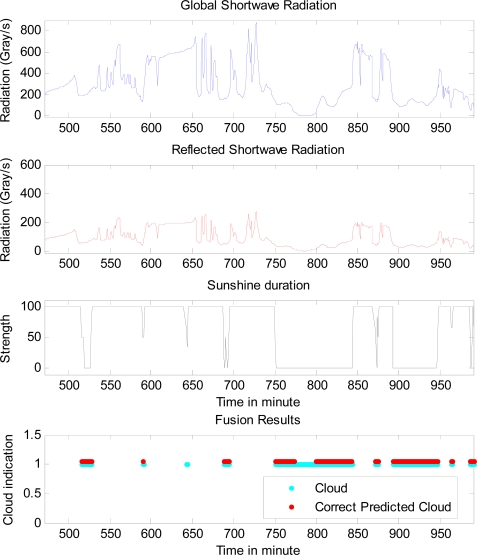
The comparison of the real cloud presence and the predicted cloud presence in a period, along with related sensor data on 13th of April.

From the tables it can be seen that Dempster-Shafer data fusion can increase correct rate of cloud prediction for all the cases, comparing to the original single sensor prediction. It can also be seen that the fusion reduces the unknown rate of cloud prediction as well. For example, for the data on 13th April, the improvement of correct rate of cloud prediction is about 10% for cloud predictor 1 (*i.e.*, 47.5% comparing to 37.8% or 38%), and about 14% for predictors 2 and 3 (*i.e*., 53.3% comparing to 39.2% or 39.4%, and 53.9% comparing to 39.8% or 40.1%). The improvement of unknown rate of cloud prediction is about 20% for predictor 1 (*i.e*., 38.7% comparing to 58.4% or 58%), and about 23% for predictors 2 and 3 (*i.e*., 31.9% comparing to 55.1% or 54.5%, and 31.1% comparing to 53.7% or 52.9%). Figure 3 gives the comparison of the real cloud presence and the predicted cloud presence in a period, along with related sensor data.

For the data on 14th April, the improvement of correct rate of cloud prediction is about 10% for cloud predictor 1 (*i.e*., 71.2% comparing to 61.2%), and about 10% for predictors 2 and 3 (*i.e.*, 72.1% comparing to 62.5%, and 72.3 % comparing to 62.4%). The improvement of unknown rate of cloud prediction is about 13% for predictor 1 (*i.e*., 23.7% comparing to 36.6%), and about 12% for predictors 2 and 3 (*i.e*., 22.6% comparing to 34.7%, and 22.9% comparing to 35%).

**Figure 4.**
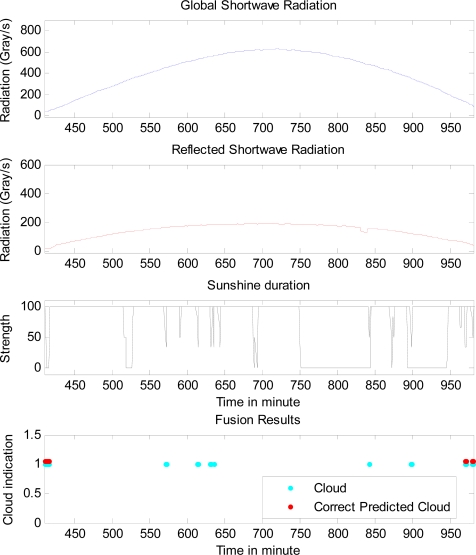
The comparison of the real cloud presence and the predicted cloud presence in a period, along with related sensor data on 29th of April.

For the data on 29th of April, Dempster-Shafer data fusion doubles the correct rate of cloud prediction and cut the unknown rate of cloud prediction about half, although the total correct rate of cloud prediction is still very low. This lack of prediction accuracy comes from the very low occurrence of cloud as shown in Figure 4.

In summary, our experiments have indicated that the proposed data fusion approach has improved cloud prediction performance greatly. It is noted that the performance of the data fusion is largely affected by the predictor. In particular, among the proposed three predictors, predictor 3 has achieved best performance (up to 72.3% correct rate of cloud prediction and as low as 22.9% unknown rate of cloud prediction), followed by predictor 2 and 1.

## 5. Conclusions

We have introduced a novel data fusion approach in a multiple radiation sensor environment using Dempster-Shafer evidence theory. The methodology is used to predict cloud presence based on the inputs of radiation sensors. Different radiation data have been used for the cloud prediction. The potential application areas of the algorithm include renewable power for virtual power station where the prediction of cloud presence is the most challenging issue for its photovoltaic output. The algorithm is validated by comparing the predicted cloud presence with the corresponding sunshine occurrence data that were recorded as the benchmark. Our experiments have indicated that the proposed data fusion approach has improved cloud prediction performance greatly. Comparing to the approaches using individual sensors, the proposed data fusion approach can increase correct rate of cloud prediction by ten percent, and decrease unknown rate of cloud prediction by twenty three percent. The performance of the data fusion is largely affected by the predictor. In particular, among the proposed three predictors, predictor 3, which has considered time shifting sensor data, has achieved best performance (up to 72.3% correct rate of cloud prediction and as low as 22.9% unknown rate of cloud prediction), followed by predictor 2 and 1 where less or none time shifting sensor data have been considered.

## Figures and Tables

**Table 1.** Intersections and products of two sensor’s basic belief mass.

Sensor 1	{c} = 0.2	{s} = 0.6	{u} = 0.2
Sensor 2			
{c} = 0.5	{c} = ***0.1***	{Φ} = **0.3**	{c} = ***0.1***
{s} = 0.4	{Φ} = **0.08**	{s}= 0.24	{s} = 0.08
{u} = 0.1	{c} = ***0.02***	{s} = 0.06	{u} = **0.02**

**Table 2.** Correct rate of cloud prediction (*C_c_*) and unknown rate (*U*) for individual sensors with different predictors.

Date	Predictor 1		Predictor 2		Predictor 3	
	Sensor 1	Sensor 2	Sensor 1	Sensor 2	Sensor 1	Sensor 2
	*C_c_* &*U* (*%*)	*C_c_* & *U* (*%*)	*C_c_* & *U* (*%*)	*C_c_* & *U* (*%*)	*C_c_* & *U* (*%*)	*C_c_* & *U* (*%*)
13th	37.8 & 58.4	38 & 58	39.2 & 55.1	39.4 & 54.5	39.8 & 53.7	40.1 & 52.9
14th	61.2 & 36.6	61.2 & 36.6	62.5 & 34.7	62.5 & 34.7	62.4 & 35	62.4 & 35
28th	32.7& 56.7	34.5 & 52.3	35.3 & 50.5	37.6 & 45.7	35.4 & 49.7	38 & 44.6
29th	4.4 & 47.6	5.8 & 36	4.4 & 47.2	5.9 & 35.3	4.4 & 47.3	5.9 & 35.3

**Table 3.** Correct rate of cloud prediction (Cc) and unknown rate (U) for fused sensors with different predictors.

Date	Fusion		
	Predictor 1	Predictor 2	Predictor 3
	*C_c_*(*%*) & *U*(*%*)	*C_c_*(*%*) & *U*(*%*)	*C_c_*(*%*) & *U*(*%*)
13th	47.5 & 38.7	53.3 & 31.9	53.9 & 31.1
14th	71.2 & 23.7	72.1 & 22.6	72.3 & 22.9
28th	49.4 & 28.2	55 & 23.4	56.1 & 22.7
29th	8 & 20.1	8.8 & 18.3	10 & 17.6
